# Modern Computational Techniques for the HMMER Sequence Analysis

**DOI:** 10.1155/2013/252183

**Published:** 2013-09-03

**Authors:** Xiandong Meng, Yanqing Ji

**Affiliations:** ^1^Genomics Division, Lawrence Berkeley National Laboratory, Berkeley, CA 94720, USA; ^2^Department of Energy, Joint Genome Institute, Walnut Creek, CA 94598, USA; ^3^Department of Electrical and Computer Engineering, Gonzaga University, Spokane, WA 99258, USA

## Abstract

This paper focuses on the latest research and critical reviews on modern computing architectures, software and hardware accelerated algorithms for bioinformatics data analysis with an emphasis on one of the most important sequence analysis applications—hidden Markov models (HMM). We show the detailed performance comparison of sequence analysis tools on various computing platforms recently developed in the bioinformatics society. The characteristics of the sequence analysis, such as data and compute-intensive natures, make it very attractive to optimize and parallelize by using both traditional software approach and innovated hardware acceleration technologies.

## 1. Introduction

At the beginning of the 21st century, an explosion of information was discovered from the living organisms, especially in areas of molecular biology and genetics. The focus of bioinformatics deals with this flood of information, which comes from academy, industry, and government labs, and turning it into useful knowledge. Bioinformatics is important to a virtually unlimited number of fields. As the genetic information being organized into computerized databases and their sizes steadily grow, molecular biologists need effective and efficient computational tools to store and retrieve the cognate information such as biological information from the databases, to analyze the sequence patterns they contain, and to extract the biological knowledge the sequences contain.

The field of bioinformatics computing is advancing at an unprecedented rate. For people working with genomics and high-throughput sequencing data analysis, it is a serious challenge to analyze the vast amounts of data coming from the next generation sequencing (NGS) instruments. For example, there were approximately 126, 551, 501, and 141 bases in 135, 440, and 924 sequence records in the traditional GenBank divisions as of April 2011 [1]. The tendency is likely only to be reinforced by new generation sequencers, for example, Illumina HiSeq 2500 generating up to 120 Gb of data in 17 hours per run [2]. Data in itself is almost useless until it is analyzed and correctly interpreted. The draft of the human genome has given us a genetic list of what is necessary for building a human: approximately 35,000 genes. For a genome as large as the human genome, it may take many days of CPU time on large-memory, multiprocessor computers to analyze. To handle this much data, computational strategies are important to tackle this vital bottleneck, which can aid scientists in the extraction of useful and important biological data.

Algorithms for biological sequence comparison can be categorized into two groups: exhaustive and heuristic. Exhaustive algorithms based on dynamic programming give optimal solutions, and well-known search algorithms like the Smith and Waterman [3], Needleman and Wunsch [4], and HMM (Hidden Markov Models) [5] are of the dynamic kind. Examples of heuristic algorithms are the BLAST [6], FASTA [7], and Feng and Doolittle [8] algorithms. Heuristic algorithms are statistically driven sequence searches and alignment methods, and not as sensitive as the exhaustive algorithms such as the Smith and Waterman algorithm and HMM.

An overview given in this paper concentrates on the computational capabilities and achievable performance of the systems discussed. To do full justice to all aspects of present high-performance implementations of the sequence analysis, we should consider their I/O performances and optimization as well. The methods we obtained from the entries of the individual implementations may be useful to many other bioinformatics applications. We believe that such an overview is useful for those who want to obtain a general idea about the various means by which these implementations achieved at high performance and high throughput with the most recent computing techniques. 

Although most computer architecture and parallelization terms are familiar to many technical readers, we think it is worthwhile to give some concise information about high-performance computer architectures and the various processors employed in these research works in Section II, in order to better appreciate the systems information given in this paper.

The majority of parallel systems are computing clusters of Reduced Instruction Set computing (RISC) based symmetric multi-processing (SMP) nodes which in turn are connected by a fast network. Shared and distributed-memory SIMD (Single Instruction Multiple Data) and MIMD (Multiple Instruction Multiple Data) implementations which are described according to their macroarchitectural class are discussed in Section 3.

The bioinformatics computing research is a very dynamic field and is especially true for the hardware-accelerated cluster world that has emerged at a tremendous rate in the last few years. The amount of research work that is related to hardware-accelerated biocomputing has boomed correspondingly. We comment on hardware characteristics and their position relative to other methods in Section 4, such as GPUs (Graphics Processing Units), FPGAs (Field Programmable Gate Arrays), and CELL BE (Cell Broadband Engine) Architecture. We have discussion and draw conclusion in Section 5 and Section 6, respectively.

## 2. Background

### 2.1. Introduction to Hidden Markov Models (HMMs)

An HMM is a statistical modeling method that has been widely used in the area of computational biology since the early 1990s. HMMs were originally used in speech recognition and then borrowed to predict protein structures and analyze genome sequences. 

**Figure 1:**
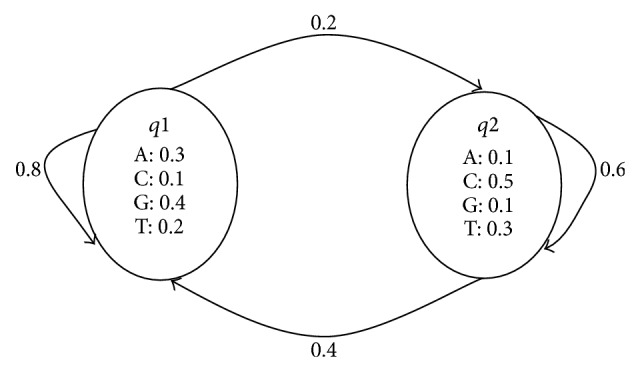
A hidden Markov model.

An HMM consists of a set of interconnected states {*q*1, *q*2, …, *qn*}. Each state can transition to another state according to state-transition probabilities {*Pi*, *j*}. *Pi*, *j* is defined as the probability that state *qi* at time *t* transmits to state *qj* at time *t* + 1. One state can also “emit” a set of symbols (residues) {*v*1, *v*2, …, *vn*} based on emission probabilities {*Pivk*}. *Pivk* represents the probability that state *qi* emits symbol *vk*.  Figure 1 gives a simple HMM that has two states. All the possible state transitions and their associated transition probabilities are shown by arrows in the figure, where the set of symbols (i.e. {A, C, G, T}) that can be emitted by that state as well as the emission probability for each symbol is shown in the state boxes (Figure 1).

**Figure 2:**
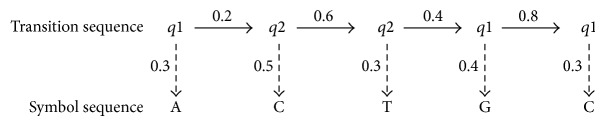
An example of a possible transmission sequence and symbol sequence.

HMMs can be viewed as a generative model which can generate sequences. Starting from an initial state, a sequence of states can be generated by moving from state to state according to the state transition probabilities. Then, a symbol sequence can be produced by allowing each state to emit symbols according to its emission probability distribution. The following gives an example of a possible transmission sequence and symbol sequence for the HMM in Figure 2.

Given this example, we can easily calculate the probability of the above state transition sequence. If we assume that the probability of being in state *q*1 at time *t* = 0 is *Pq*1(0) = 0.7 (i.e., the initial state), we can get

(1) P[q1  q2  q2  q1  q1]=0.7×0.2×0.6×0.4×0.8=0.02688.

We can also calculate the probability of the above symbol sequence given the above transition sequence:

(2) P[(ACTGC) ∣ (q1  q2  q2  q1  q1)]  =0.3×0.5×0.3×0.4×0.1=0.0018.

The probability of obtaining the above transition sequence and the above symbol sequence is

(3) P[(ACTGC)∧(q1  q2  q2  q1  q1)]  =0.02688×0.0018=4.8384×10−5.

One can see that HMMs provide a mathematical model for “linear” problems like sequences or time series. In practice, only the output symbol sequence is observable and the state transition sequence is “hidden” from us. Hence, we must infer the state sequence by given observed sequence data (e.g., DNA sequences). Please also note that while an HMM has a finite number of states, it can generate infinite number of possible sequences.

**Figure 3:**
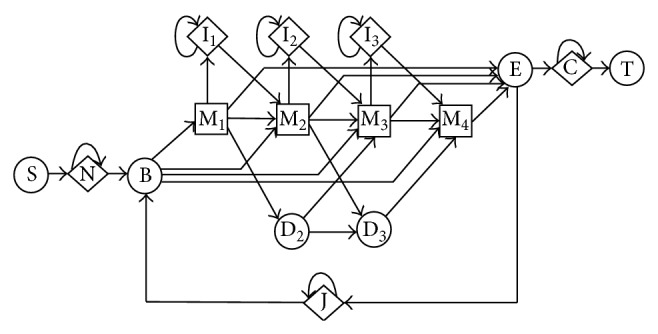
Plan 7 HMM model architecture.

HMMs have been successfully employed to represent profiles of multiple sequence alignment in computational biology [5]. A “profile” is defined as “a consensus primary structure model consisting of position-specific residue scores and insertion or deletion penalties” [20]. Various HMM architectures have been proposed to model a profile in the literature [21]. While traditional pairwise alignment strategies such as Smith-Waterman [3], BLAST [6], and FASTA [7] use position-independent scoring, these HMM-based models can capture position-specific alignment information. The “Plan 7” model [21] is a representative profile of HMM architecture in Figure 3. A multiple sequence alignment of homologous protein sequences can be precisely represented by such a model.

There are three major types of states in this architecture: matching states (M), insertion states (I), and deletion states (D). Arrows were used to indicate transitions between different states with associated transition probabilities. The matching states were used to model the distribution of residues allowed in the columns of a multiple sequence alignment. That is, each state has an emission distribution that reflects the frequency of each residue observed in the corresponding column of that alignment. One can see that each matching state can transit to another matching state and different types of other states (e.g., I, D) according to its transition probabilities. An insertion state exists between each pair of matching states. It not only transits to a matching state but also transmits to itself which allows one or more symbols to be inserted. Each insertion state also carries an emission distribution. Since there exist 20 different amino acid residues for protein sequences, each matching state or insertion state carries a set of 20 emission probabilities. A deletion state allows a column to be skipped and it emits nothing. The Plan 7 model starts from state B and ends at state E. In addition, it contains five special states (i.e., S, N, C, T, and J) that deal with alignment specific features (e.g., global or local alignment).

Given a profile HMM like the Plan 7 model, researchers are typically interested in three problems: (1) how are different parameters of the model learned given some observed sequence data? That is, given the architecture or topology of a HMM and observed data, we want to find the optimal model that maximizes *P* (*observations*∣*HMM*); (2) given an existing optimized HMM and an observed sequence, what is probability that the model produces that sequence (i.e., *P* (*sequence*∣*HMM*))? (3) what is the most likely state sequence that the HMM would use to generate a sequence? That is, we want to find the optimal state sequence *q*1, *q*2, …, *qn* such that *P* (*q*1, *q*2,…, *qn*∣*sequence*, *HMM*) is maximized.

 HMMER [21, 22] is a software tool that relies on the Plan 7 model. It consists of a collection of programs that can be employed to solve the above three problems.

### 2.2. Accelerating Platform

Due to the huge amount of genomic sequence data, exploring acceleration techniques becomes necessary in bioinformatics computing such as sequence alignment and sequence database searches. There are various accelerating platforms that allow performing multiple computations in parallel so as to reduce the total computing time. An accelerating platform typically contains both hardware and software. While the hardware includes processors and memory, the software consists of computer programs and data stored in memory. In addition, special-purpose hardware can be specifically designed for a particular type of bioinformatics computing (e.g., sequence searches). 

**Figure 4:**
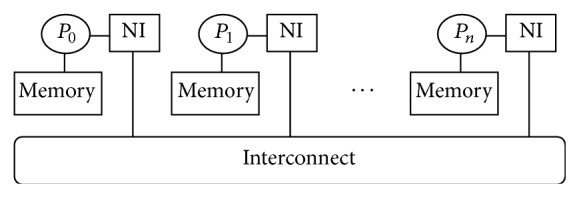
A computer cluster with *n* nodes.

Parallelism can be achieved both within one computer and among a group of computers. Within one computer, instruction level parallelism has been exploited at both compile time and runtime. Modern compilers can detect independent instructions and pack them together in VLIW (Very Long Instruction Word) for parallel execution [23]. During runtime, CPUs (Central Processing Units) can reorder the execution of instructions such that those independent instructions can be executed simultaneously. Moreover, Intel's HTT (Hyper-Threading Technology) or SMT (Simultaneous MultiThreading) supports parallel execution of multiple instructions from different hardware threads-running programs' control flows. In recent years, the multi/many-core computer architecture allows several processing units to be built inside a single CPU chip where each core can host multiple threads. Furthermore, multiple CPU chips can be connected inside a single box, called SMP (Symmetrical Multiprocessors), for processor level parallelism. Also, a group of computers can be connected through fast networks and work as a virtual supercomputer. Such cluster computing is becoming more and more popular.  Figure 4 shows a computer cluster containing n independent nodes. Each node has its own memory and cache but cannot directly access another processor's memory. Each node also has an NI (Network Interface) for all communication and synchronization. Please note that a node can be either a single-CPU computer or an SMP.

To effectively and efficiently utilize various hardware resources at different granularity levels, software needs to decompose data and programs, map them onto multiple processing units, and support communication in order to coordinate different subtasks. The shared address space programming paradigm (i.e., multithreading) is a widely used approach on single CPU machines or shared memory multiprocessors [23]. With this programming paradigm, programmers develop multiple threads that process different data sets. Communications among different threads are implicit and achieved through global variables. POSIX (Portable Operating System Interface) Threads [24] and OpenMP (Open Multi-Processing) [25] are popular software packages that support multithread programming. The major advantages of this paradigm are programmability and flexibility. However, it cannot be directly applied on clusters. On distributed memory machines or clusters, the message passing programming paradigm is more effective and commonly used. With this approach, multiple programs are developed and executed concurrently on different computers. Each program runs independently unless it needs to communicate with another program in order to share and synchronize data. If the overhead of such communications can be under control, this approach will utilize the otherwise idle processors in multiprocessors or idle computers in clusters. MPI (Message Passing Interface) [26] is a representative library for this paradigm. 

Using MPI or POSIX to accelerate sequence alignment tools like HMMER requires high-performance multiprocessor computers or large-scale computing clusters. These computing systems are often very expensive. An alternative approach is to design special-purpose hardware that can operate on normal computers. Since such hardware is specifically tailored for the sequence alignment problem, good performance can be easily achieved. FPGA has been used to build special-purpose hardware for accelerating HMMER [19, 27, 28]. An FPGA is an integrated circuits that contains a lot of programmable interconnected “logic blocks.” It can be configured to implement various algorithms using a hardware description language. The advantage of this approach is that it can easily achieve good performance since the hardware is especially tailored for the sequence alignment problem. Its disadvantages include high initial design cost and programming complexity of the hardware description language. In addition, the special-purpose hardware might not be employed to solve other problems. GPU cards have also been applied to accelerate HMMER as well as other sequence alignment algorithms [15, 29]. Today's commodity GPU cards can provide tremendous memory bandwidth and computational horsepower. Moreover, software tools such as General-Purpose computation on GPU, NVIDIA's CUDA (Compute Unified Device Architecture) [30] and Apple's OpenCL [31] have made parallel programming on GPUs much easier than before.

### 2.3. Measuring Performance

There are various metrics, for example, speedup, efficiency, and cost, which are used to measure the performance of a certain parallel program. No single method is usually preferred over another since each of them reflects certain properties of the parallel code. A straightforward measure of the parallel performance would be the ratio of the execution time on a single processor (the sequential version) to that on a multicomputer. The speedup of any parallel computing environment obeys Amdahl's Law [32].

Amdahl's law describes the relationship between the expected speedup of accelerated implementations of a program relative to the original nonaccelerated program. It states that the maximum expected performance improvement to be gained from using a faster mode is limited by the fraction of the time the faster mode cannot be applied to. For example, assume that we have a program that needs 10 hours using a single-processor computer and a particular portion of 1 hour cannot be accelerated. That is, only the remaining portion of 9 hours can be accelerated, either using multiple processors or special-purpose hardware. Intuitively, no matter how many processors or hardware units we employ to accelerate this program, the overall execution time cannot be less than the critical 1 hour. That is, the overall speedup is limited by 10/1 = 10x.

Theoretically, Amdahl's law is depicted by the following equation:

(4) $$\begin{array}{c} \text{overall}\;\text{speedup}=\frac{1}{\left(1-P\right)+\left(P/S\right)}, \end{array}$$

where *P* represents a fraction of a program that can be accelerated and *S* is the corresponding speedup of the enhanced portion. In the above example, *P* is equal to 9/10 = 0.9. If we assume the portion of 9 hours can be mapped onto 4 processors or hardware units and executed simultaneously, then *S* is 4, assuming no extra overheads are introduced. In this case, the overall expected speedup is

(5) $$\begin{array}{c} \text{overall}\;\text{speedup}=\frac{1}{\left(1-0.9\right)+\left(0.9/4\right)}=3.08. \end{array}$$

One can see that, given limited hardware resources (e.g., 4 processors), the most important thing for accelerating a program is to detect the time-consuming portion that can be parallelized or accelerated. Note that the acceleration process often introduces additional overhead such as data transfer to and from the external hardware accelerators, data redecomposition and reformatting, and communication among different hardware components. Therefore, the actual achievable speedup is usually less than the theoretical value obtained by Amdahl's law.

## 3. Software Accelerated HMMER

Various software approaches have been applied to accelerate the original HMMER programs which were developed by Eddy and his coworkers [21, 22]. Most of these approaches focus on accelerating one or more of the three programs: hmmpfam, hmmsearch, and hmmcalibrate. hmmpfam is used to search a database of profile HMMs against a given query sequence. hmmsearch is used to perform sequence database searches that matches an input profile HMM. hmmcalibrate takes a profile HMM and determines its statistical significance parameters which make the database search more sensitive. In the literature, various strategies have been applied to modify and parallelize these programs in order to take advantage of different levels of commodity hardware components and achieve much better performance.

### 3.1. Instruction Level Parallelism

Modern general purpose processors often have 16-byte-wide registers that can hold and process multiple data items within one single instruction. This SIMD (Single Instruction Multiple Data) technique can be used to explore fine-grained instruction level parallelism. A variety of commodity CPUs such as AMD Opteron, AMD Turion 64, Intel Xeon, and Intel Core Solo/Duo provide SIMD instructions and corresponding large registers. These instructions have a great potential to accelerate sequence analysis in biological computing due to its data-intensive nature.

SSE2 (Streaming SIMD Extensions 2) instructions [33] from Intel are a representative set of SIMD instructions that extends Intel's previous MMX (MultiMedia eXtension) and SSE technologies. They provide a series of packed integer operations and double precision floating point operations that work on 128-bit data. Walters and his colleagues [9] have attempted the use of SSE2 instructions to accelerate HMMER. Since reimplementing the entire HMMER program using SSE2 instructions is costly and time consuming, they only focused on the innermost loop of the Viterbi function used by both hmmpfam and hmmsearch when performing a search. That loop consumes more than 50% of the execution time for both programs. Moreover, only additions and maximum value selections over 32-bit integers are included in this short segment of code. Since SSE2 instructions operate on 128-bit data, four integer operations are allowed to be performed in parallel. Therefore, the ideal speedup of this loop is 4x. According to the Amdahl's law introduced in Section 2.3, the ideal overall speedup would be 1/((1 − 0.5) + 0.5/4) = 1.6.

However, it is very difficult to achieve the above speedup because there are several types of overhead during the reimplementation of the code of that loop. First, there exist interiteration dependencies in the loop. That is, operations in the current iteration need results from the previous iteration. Therefore, the code in the loop must be rearranged and split into several loops in order to remove the dependencies. Otherwise SSE2 instruction cannot be applied. This introduces additional overhead. Second, the SSE2 does not have direct max/min instructions which are required in order to reimplement the loop. The max/min operations must be implemented using the existing instructions, which introduces further overhead. Third, extraoperations, such as data alignment and the moving of data into the 128-bit registers, also bring in additional overhead.

Experiments were performed on a 2.66 GHz Intel Xeon processor with 2.5 GB of memory. Both hmmpfam and hmmsearch were tested using various samples from the Pfam and nr databases. While the Pfam database contains many multiple sequence alignments and HMMs represent different protein families [34], the nr database is a sequence database from NCBI (National Center for Biotechnology Information) [35]. The resultant overall speedups range from 1.2 to 1.3.

The major advantage of using SSE2 to accelerate HMMER is that it does not require new hardware upgrades and the cost is almost free. But the critical segment of code that consumes most of the execution time must be identified. If that segment of code is short, reimplementing the code is trivial and does not need much development time. However, its disadvantage is that the modified codes from C to intrinsic/assembly are not portable but architecture dependent. That is, moving the modified code from one platform to another requires reimplementing the code again for the new platform.

### 3.2. Shared Memory Parallelism

While SIMD instructions exploit instruction-level parallelism within one CPU, in order to take advantage of multiple CPUs or CPUs with multiple cores in a computer system, users must develop multithreading programs. As a widespread programming model, multithreading allows a process to generate multiple software threads that share the same virtual address space. These threads inherit many resources from the hosting process and, at the same time, have their own stacks and registers. Programmers need to partition source code or data into different threads that will be mapped onto different hardware units. Then, these threads execute concurrently and finish their own jobs in parallel. 

One of the most widely used thread libraries is Pthread (POSIX Threads) [24]. To develop multithreaded programs, users have to insert library calls to create, delete, and synchronize threads. The created programs can be compiled by most C compilers. OpenMP [25] also supports multi-thread programming in C/C++ and FORTRAN. It uses directives to implement parallelism instead of explicitly handling threads. OpenMP compilers will translate those directives to Pthread functions without the programmer's involvement. Thus, compared to Pthread, OpenMP is easier to use. However, it requires OpenMP compliers to compile programs and it is often difficult to deal with irregular problems, such as problems inherent in sparse and unstructured computations.

The original HMMER package provides Pthread implementations of three programs: hmmsearch, hmmpfam, and hmmcalibrate. To run these programs on a multiprocessor machine, users only need to enable Pthread support by passing the “—enable-threads” option to the  ./configure script. Srinivasan et al. [10] in the Intel Corporation reimplemented the hmmpfam program using OpenMP directives and tested its performance on various Intel ×86 architecture-based shared memory multiprocessor (SMP) systems. With 600 HMM models and 250 sequences, the speedup is 3.36 on a four-processor SMP system (2.0 Hz). Its performance was also compared on two SMP systems that both have sixteen x86 processors. But one of the systems has 8 GB of memory and its processors are 3.0 GHz. The other system has 16 GB of memory and its processors are 2.2 GHz. The speedup of the former system is 14 whereas the speedup for the latter system is about 10.3. This comparison indicates that the multithreaded HMMER hmmpfam program is more sensitive to CPU speed than memory size.

Another multi-threading implementation of the hmmpfam program was done by Zhu et al. [11]. They reimplemented hmmpfam on EARTH (Efficient Architecture for Running Threads)—an event-driven fine-grain multithreaded program execution model. Through its Threaded-C language (an extension of C), EARTH allows multithreaded programs to run on distributed memory systems like clusters [36]. The EARTH-based multithreaded hmmpfam achieved about 30 speedups on a cluster of 18 nodes, each containing two 1.4 GHz AMD Athlon processors. On a much larger cluster that consists of 128 nodes, each with two 500 MHz Pentium III processors, the achieved speedup is as high as 222.8 for a data set containing 50 HMM profiles and 38,192 sequences.

### 3.3. Distributed Memory Parallelism

While SSE2 allows users to exploit instruction level parallelism within one single processor, distributed memory parallelism provides opportunities to execute programs in parallel across different computers. A group of processes can be started simultaneously and mapped onto multiple machines, each of which accomplishes a subtask. Since processes do not share the address spaces, communication approaches such as MPI [26] and PVM (Parallel Virtual Machine) [37] must be utilized across machines in order to send/receive messages and coordinate and manage different tasks. 

Cluster computing has become very popular in the field of high-performance computing. Currently, most of the Top500 supercomputers are labeled as “clusters” [38]. A cluster can be viewed as a parallel computer system that consists of an integrated collection of independent “nodes.” These nodes can work together closely but each of them can perform independent operations and may be derived from products developed for other standalone purposes. With a fast network, common users can easily build their own clusters. There exist several variations of cluster computing such as Internet Computing, Grid Computing, and Cloud Computing. These variations simply emphasize different aspects or levels of cluster computing. 

Clusters are very suitable for implementing distributed memory parallelism and have been widely used in bioinformatics computing. It typically takes a master-slave model where a master node distributes subtasks to different slave nodes, and slave nodes do the real computing work and return results to the master node. Under the supervision of Professor Vijay Pande at Stanford University, the folding@home project [39] is such an example where volunteer computers form one of the largest clusters in the world. A volunteer computer always contacts the server (i.e., master) for protein folding work, and every time they finish their local work, they contact the server again for extra work. This large distributed computing platform allows us to do extremely challenging computation that could not be achieved before. It is particularly useful for bioinformatics computing because these problems are often computationally intensive. That is, the communication overhead over the long distance to the master node is trivial when compared to the computing time. 

The message-passing programming paradigm is essential for distributed memory parallelism. Both PVM and MPI have been adopted for implementing parallel versions of HMMER that can run on distributed clusters. The original HMMER package provides a PVM implementation of three programs: hmmsearch, hmmpfam, and hmmcalibrate [40]. In this implementation, the computation for one sequence is executed concurrently, and the master node dynamically assigns one profile to a specific slave node for comparison. Upon finishing its job, the slave node reports the results to the master, which will respond by assigning a new profile. When all the comparison regarding this sequence is completed, the master node sorts and ranks all the results it collects and outputs the top hits. Then the computation on the next sequence begins.

Walters et al. [9] reimplemented hmmsearch and hmmpfam using MPI and compared their performance to their corresponding PVM implementations. There are two major justifications for the re-implementation. First, it extends the use of HMMER to those people who prefer MPI. Second, since PVM does not truly support asynchronous sends, implementing a nonblocking, double-buffering strategy using MPI has the potential to hide communication latency and thus achieve better performance. The double-buffering scheme allows a slave node to compute on a buffer while at the same time receiving another sequence into a second buffer. The basic idea of this strategy is to overlap the computation time and communication time as much as possible. To further reduce the communication time, the researchers employed a database chunking technique to minimize the number of sends and receives. This technique allows a node to receive and work on batches of sequences simultaneously before returning its results to the master node. 

In [9] Walters et al. also combined the MPI implementation with the SSE2 implementation. This combined version of HMMER can take advantage of both the parallelism between computing nodes and the instruction level parallelism within a single workflow. The performance of three HMMER implementations (i.e., PVM, MPI, MPI + SSE2) was compared on a university cluster in which each node consists of two 2.66 GHz Intel 4 Xeon processors and different nodes communicate through 100-Mbit Ethernet. Experiments have shown that the MPI implementation outperformed the PVM implementation, and the MPI + SSE2 implementation gained even better performance. For example, the speedups of hmmsearch on 16 CPUs for the three implementations are 4.56, 5.90, and 7.71, respectively, using a 100 MB database.

### 3.4. Other Issues and Optimizations

With various software tools, parallelisms can be exploited at different levels. This can greatly improve the performance of the HMMER program. However, there are several other issues such as scalability, I/O, and quality of code that can also affect the performance of the accelerated HMMER. If these issues are not handled appropriately, the computing power of a large system cannot be fully utilized and thus the speedup is only limited. These issues and relevant optimizations are discussed below.

Scalability is a very important issue for a massively parallel system/cluster that contains hundreds or thousands of nodes. A system is considered to be scalable if its performance improves proportionally to the hardware added. The scalability of a large parallel system can be affected by many factors such as communication latency, data dependency, and organization of involved processes. Jiang and his colleagues [41] studied the techniques that can improve the scalability of the three HMMER programs (i.e., hmmsearch, hmmpfam, and hmmcalibrate) on the Blue Gene/L (BG/L) massively parallel supercomputer from IBM [42]. The MPI implementation of hmmcalibrate is found to be scalable and its scalability is up to 2,048 nodes. However, the observed scalability of hmmsearch and hmmpfam is no higher than 64 nodes; that is, no improvement was observed beyond 64 nodes. 

Bioinformatics applications like HMMER are not only computationally intensive but also highly I/O demanding. Data intensive input and output operations can become the performance bottleneck like the HMMER program, especially when large databases are searched. This “Disk Wall” problem will become worse along with the exponential growth of database sizes. Walters et al. [12] studied the I/O problems in HMMER and enhanced its MPI implementation (called MPI-HMMER) using parallel I/O and a parallel file system. In this new version (called PIO-HMMER), the database distribution mechanism was modified so that the master node, instead of directly reading data and sending them to slave nodes, only distributes sequence indexes (each containing sequence offsets and lengths) to slave nodes and slave nodes read from the database in parallel. In addition, several new optimizations were implemented to further improve the overall performance: (1) enhanced postprocessing reducing the number of messages being sent to the master node by only returning those messages resulting in hits; (2) a database chunking technique similar to the one implemented in MPI-HMMER, but with larger chunks of the database for parallel I/O; (3) asynchronous I/O for returning scores to the master node; (4) a load-balancing scheme for hmmsearch achieved by allocating database based on the lengths of sequences instead of assigning equal number of sequences to each slave node; (5) a database caching scheme similar to the one implemented by Jiang et al. [41].

Various experiments were performed on a cluster with 1056 nodes, each equipped with two 3.2 GHz Intel Xeon processors and 2 GB RAM. With parallel I/O and the above optimizations, the overall speedup achieved by PIO-HMMER is 221x for hmmsearch and 328x for hmmpfam, while MPI-HMMER is only able to achieve 55x speedup for hmmsearch and 27x speedup for hmmpfam. The performance impact of some individual optimizations (including parallel I/O) was also tested. As shown by experiments, the parallel I/O represents the single greatest performance impact of all optimizations. It improved the speedup from 42x to 190x for hmmsearch (searching a 236 state HMM against a large sequence database). The I/O problem also affects the scalability of the HMMER programs. For example, the MPI-HMMER's nonlinear scalability is only 64 nodes whereas PIO-HMMER's scalability is extended to 256 nodes. Experiments also demonstrated that both MPI-HMMER and PIO-HMMER can achieve better scalability for large data sets (i.e., large HMM database and sequence database). This is consistent with the results obtained by Srinivasan et al. who tested the scalability of an OpenMP implementation of HMMER using various datasets. Their experiments revealed that the scalability of multithreaded HMMER improves when the size of the input data increases. The background reason of this observation is that proportion of time spent in the sequential code decreases with respect to the time spent in the parallel portion when large data sets are used.

The quality of source code may affect compiler/runtime optimizations and cache misses and as a result influence the performance of a program. Waters et al. made minimal changes (e.g., removing unnecessary intermediate variables, breaking iteration dependencies) of the loop in the P7Viterbi routine and achieved 1.8x speedup for hmmcalibrate. By splitting the loop and breaking the intraiteration dependence in the Viterbi algorithm, Srinivasan et al. [10] improved the overall execution time by 10–16% for hmmpfam. All these changes, while minor, can have a big effect on the compiler optimization and result in modest performance improvements. Srinivasan et al. also attempted to reduce the number of cache misses by dynamically allocating the size of the dp_matrix used by hmmpfam so that it matches the size required by a profile HMM. On a 4-processor machine, ~16% improvement of execution time was observed by this optimization.

### 3.5. Summary

The achieved performance of different software approaches for accelerating HMMER is summarized in Table 1. Some researchers or research groups did various experiments using various database sizes or hardware configurations. In this case, only the best achieved speedup is included in the table. Please also note that the work from Jiang et al. [41] is primarily focused on scalability, and explicit speedups of their experiments were not provided. Thus, their work is not included in this table either.

Even though the achieved speedups cannot be directly compared due to the variations in experimental setups and database sizes, several observations can be made from this table. First, the overall speedup achieved by instruction level parallelism is limited since it only exploits internal parallelism within one single processor. But this approach does not require any new hardware and can be easily combined with other approaches like MPI. Second, the performance achieved by shared memory parallelism is generally moderate since the number of processors sharing the same memory is often limited. However, good performance can be obtained under the assistance of EARTH since it provides an efficient architecture for running threads on large distributed memory systems like clusters. Finally, compared with instruction level parallelism and shared memory parallelism, distributed memory parallelism has the greatest potential to achieve the best performance after carefully taking care of I/O issues and other communication bottlenecks. The disadvantage of this approach is its programming complexity. Programmers need to explicitly divide databases, map computing tasks onto different nodes, and handle various synchronizations among different tasks.

## 4. Hardware-Accelerated HMMER

As an alternative to software accelerated computing, such as shared and distributed memory computing systems, hardware-accelerated computing has been advocated in bioinformatics applications by combining powers of special-purpose hardware and existing computational resources. In this section, we give most HMMER implementation details with an emphasis on the hardware architecture in various forms of which we will discuss some general characteristics.

### 4.1. Accelerated Computing

It has become all the more clear that no one type of processor is best for all types of computation. In the recent several years, a wide range of computational hardware accelerators have been applied to bioinformatics research. The developments in this field are rapidly gaining popularity. 

Today, microprocessors are more powerful for general purpose computing, but it is still too slow to perform the HMMER profile search which is extremely data and compute intensive. The hardware structure or architecture determines to a large extent what the possibilities and impossibilities are in speeding up a computer system beyond the capability of a single CPU. Bioinformatics users never tend to content themselves with the performance of the machines they own and are continuously seeking new breakthrough to speed up the calculation. Presently there is a group of acceleration products which can deliver significant performance gains over traditional approaches on HMMER when properly deployed.

Before going on to the descriptions of the HMMER acceleration techniques, it is useful to consider some specialized computer architectures, which have been used to increase the computational performance. We follow the main trends in emerging architectures for the heterogeneous parallel systems and discuss the most recent HMM hardware implementations on the three classes of accelerators. 

GPGPUs (General Purpose computation on Graphics Processing Units) may have been invented to power video games, but today these massively parallel devices harness the computational power to perform nongraphics calculations. The latest GPU architectures provide tremendous memory bandwidth and computational horsepower, with fully programmable vertex and pixel processing units that support vector operations up to full IEEE floating point precision. 

FPGAs provide many logic blocks linked by an interconnection fabric. The interconnections can be reconfigured dynamically, thus allowing the hardware datapaths to be optimized for a given application or algorithm. When the logic blocks are full processors, the FPGA can be used as a parallel computer. 

CELL BE is a heterogeneous architecture containing one general-purpose computer and eight SIMD-based coprocessors on a single chip. By exploiting the MIMD parallelism of the coprocessors and overlapping memory operations with computations, the Cell BE has been proved to achieve impressive performance on many bioinformatics applications. 

Moreover, hardware accelerators are now used in some of the world's fastest computers. Accelerators are now themselves parallel systems. They can also be seen as a new level in hierarchical machines, where they operate in parallel with the host processors. The top supercomputers have taken advantages of heterogeneous cooperation architecture to scientific calculation in a multiuser environment. For example, in June 2009 Top 500 list, the no. 1 supercomputer was “Roadrunner” [38], which has 129,600 cores, and utilizes IBM Cell BE processors as accelerators. GPUs are used in the world's fastest supercomputer, “Tianhe-1A” [38], No. 1 in Top500 list by Nov. 2010.

HMMER has also been accelerated on other special-purpose processor, such as network processors-JackHMMer [12], which builds on the Intel IXP 2850 network processor, a heterogeneous multicore chip consisting of an XScale CPU paired with 16 32-bit microengines. A speedup of 1.82 over a P4 running at 2.6 GHz was reported.

### 4.2. GPGPU

GPUs can offer energy-efficient performance boosts to traditional processors since they contain massive numbers of simple processors, which are more energy efficient than a smaller number of larger processors. Graphics processing is characterized by doing the same operation on massive amounts of data. To accommodate this way of processing GPUs consist of a large amount of relatively simple processors, fast but limited local memory, and fast internal buses to transport the operands and results. The key to accelerating all of these operations is parallel computing, often realized by computing all pixels of a display or all objects in a list independently. 

The growing popularity of GPU-based computing for nongraphics applications has led to new interfaces for accessing GPU resources. A major challenge in the evolution of GPU programming involves preserving GPU performance levels and ease of use while increasing the generality and expressiveness of application interfaces. With improving programming models, NVIDIA's CUDA [29] allows programmers to write data-parallel applications for GPUs at the “kernel” level by specifying what operations take place on an individual data element. Newest technique by the Portland Group offers Fortran and C accelerator compilers [43] to accelerate the existing high-level standard-compliant programs for the CUDA-enabled NVIDIA GPUs by adding OpenMP-like compiler directives.

It is motivated by GPGPUs' enhanced programmability, attractive cost/performance ratio, and incredible growth in speed. Today they are being pressed into high-performance bioinformatics computing. For accelerated HMM search, a considerable amount of research made the use of a GPU for nongraphics high-performance computing more interesting.

In the paper [15], GPU-HAMMER was implemented by taking advantage of NVIDIA 8800 GTX Ultra GPUs with 768 MB RAM. The 8800 GTX Ultra is composed of 16 stream multiprocessors, each of which is itself composed of 8 stream processors for a total of 128 stream processors. It can maintain 4,096 active threads and all threads run in parallel on a single GPU with each operating on its own sequence. In order to achieve the best performance, it requires to presort the sequence database by length. Thus, it is able to achieve a nearly 7x performance over the unsorted database. When the *hmmsearch* codes were targeted to the 8800 GTX Ultra, a variety of optimizations were implemented including database-level load balancing memory layout and coalescing, loop unrolling, and shared/constant memory use. This study achieved up to 38x speedup. 

Ganesan et al. [16] redesigned the *hmmsearch* program to extract data parallelism out of the serializing data dependencies using 1 and 4 Tesla C1060s. The highlight of this work is that each GPU thread block operates on individual sequences and writes the cost of decoding to the global memory independently. Thus there is no need to sort the sequences compared to [15]. They reported that the time grows linearly with the HMM module size and scales linearly with the number of GPUs. This work showed a speedup of 5x-8x over GPU-HMMER [28]. With 4 Tesla C1060 boards, it could achieve 100 + x speedup compared to a serial implementation on an AMD Opteron at 2.33 GHz.

### 4.3. FPGA-Based Accelerators

An FPGA is an array of logic gates that can be reconfigured to fulfill user-specified tasks. In this way we can design special purpose functional units that may be very efficient for some specific purpose. In addition, FPGA allows easy upgrading and users to explore the applicability of its reconfigurable nature to various scientific problems compared to application-specific hardware. Moreover, it is possible to configure multiple FPGA boards that work in parallel. Theoretically, FPGAs may be good candidates for the acceleration of many bioinformatics applications. In general, excellent results have been reported in HMM searching. 

Because of their versatility it is difficult to specify where they will be most useful. The clock cycle of FPGAs is low as compared to that of present CPUs: 100–550 MHz which means that they are very power effective. To program FPGAs, there are now two industry standard hardware description languages, VHDL (Very high speed integrated circuit Hardware Description Language) [44] and Verilog [45]. Vendors, like Xilinx [46] and Altera [47], provide runtime environments and drivers that work with Linux as well as Windows. 

Oliver et al. [18] presented an FPGA solution that implements a full plan 7 model. Instead of computing the Viterbi algorithm on one dataset at a time, they aligned query/subject in separate processing elements (PEs) they designed in Verilog. Their design assumes that the same profile HMM has to be aligned to different sequences. All PEs are synchronized to process the same HMM state in every clock cycle. The system is connected to the HMMer software running on the host system via an USB port. The host software performs load/store FPGA and postprocess relevant hits. This strategy outperforms the sequential implementation on a desktop for both *hmmserch and hmmpfam *by one to two orders of magnitude.

Another recent research [19] proposed a systolic-array-based implementation of plan 7 HMM on FPGAs with a parallel data providing unit and an autorecalculation unit. A speedup of 56.8 with 20 PEs on a Virtex-5 board was obtained compared to an Intel Core 2 Duo 2.33 GHz CPU. 

MIP-enabled cluster could achieve excellent performance and cluster utilization of hmmsearch. Walters et al. [13] described a hybrid implementation of the HMM search tool. Combining the parallel efficiency of a cluster with one or more FPGA cards can significantly improve the HMMER's *hmmserch *functionality, in some cases achieving near linear speedup. The system is not just designed for running individual FPGA, but for scaling codes on a FPGA cluster. 

### 4.4. CELL BE Accelerators

The Cell BE was jointly designed for video gaming industry by IBM/SONY/TOSHIBA. One CELL BE [17] contains two different types of processors: one 64-bit PPE (PowerPC Processor Element) and eight SPEs (Synergistic Processing Elements), all running at a clock speed of 3.2 GHz and theoretical peak performance of 204.8 GFLOPS for single precision. PPE acts a controller for the eight SPEs, which handle most of the computational workload. Each SPE consists of a SPU (Synergistic Processing Unit) and a MFC (Memory Flow Controller). The SPU is a RISC processor with 128 128-bit SIMD registers. In addition, a high-speed memory controller and high-bandwidth bus interface are all integrated into one chip.

The nature of HMM, all-to-all comparison, has most potential to gain benefits from porting the entire program to a heterogeneous multicore processor like the CELL BE. Each query search is completely independent and thus can be performed in parallel across the 8 SPUs of the Cell Processor. Sachdeva et al. [17] implemented the computationally expensive kernel *viterbi* on the SPUs and run the reminder of the code on the PPE. Unfortunately, their preliminary implementation only worked on a single sequence being compared against a single HMM on a single SPU. However, it still showed about 3.5x faster than a 2.4 GHz dual core Opteron processor. Potentially, the CELL BE is not used as a screen platform, but also as a complete computing engine with large performance benefits. 

### 4.5. Summary

The performance of hardware acceleration is summarized in Table 2 based on the peak performance that the hardware can achieve. Note that there are no standard HMM benchmarks and the reported performance was done by individual research group using various databases and HMM models.

We have discussed the various strategies used in utilizing parallel power that accelerator offers. Porting an application to an accelerator requires reworking the code. For example, running an application efficiently on a hardware accelerator often requires keeping the data near the device to reduce the computing time taken up with moving data from the CPU or memory to the accelerator. Also, a programmer must decide how to collect results from the accelerator back into the CPU program. This is very cumbersome for the average programmer as one not only has to explicitly define such details as the placement of the configured devices but also the width of the operands to be operated on and so forth. 

The goal of hardware acceleration or hybrid computing is to reduce the search time from hours to minutes. Heterogeneous implementation utilizes a mix of hardware accelerator and compute nodes to achieve the excellent performance over that of a single node and accelerator only implementation. The results show that integrating the parallel efficiency of a cluster with one or more hardware accelerators can significantly increase performance for even the compute/data intensive HMM searches. Obviously, a hybrid cluster would be a smart choice to offer bioinformatics users access to a technology that is positioned to change how many traditional applications are written.

## 5. Discussion

In this paper, we selected HMMER, a scientific application from the domain of bioinformatics, to evaluate a variety of its implementations by comparing their performance with corresponding sequential, software, and hardware solutions. We have reviewed the above exploratory work on the use of modern computing architectures, ranging from chip level multithreading, multicore architectures, clusters, and grid computing to special architectures. 

First of all, we emphasized several observations that were verified by various experiments and the techniques might be extended to other bioinformatics applications. (1) The CPU speed has a bigger impact on the multithreaded hmmpfam program than on memory size. This observation can direct us to select appropriate hardware configurations so as to get better performance and also provide useful implications for bioinformatics computing since many bioinformatics applications are compute intensive. (2) The scalability of both MPI and multithreaded implementations of the HMMER program is sensitive to the size of the input data. The reason is that the larger the data set, the more time is spent in the parallel portion of the program. Thus, a better speedup can be obtained with respect to the Amdahl's law. This observation implies that the performance of parallel version of HMMER including both shared memory and distributed memory parallelism is related to the size of input data set. (3) Experiments on the MPI implementation of both hmmpfam and hmmsearch showed that, relatively speaking, hmmpfam is more I/O-bound while hmmsearch is more compute bound. This observation implies that performance is often application dependent. Thus, analyzing each application and carefully handling its performance bottleneck are also very important in bioinformatics computing. 

Second, clock frequencies of chips are no longer increasing, so all future improvements in computer speed will come from parallelism. Though many bioinformatics applications are parallelizable like HMMER, parallelism is usually not explicitly expressed in their original codes. Users interested in accelerating a bioinformatics application are required to either develop software to distribute data and manage parallel jobs or modify existing codes to make sure of libraries or hardware that facilitate distributed computing.

Next, the long-term goal of bioinformatics computing is to design a system that is a more efficient, much less expensive and has an immediate impact on the world's energy consumption. Bioinformatics should stand at the front of green computing to reduce energy consumption compared to other commercial systems. In comparison to general purpose CPUs, hardware accelerators all are very power effective. Sometimes orders of magnitude when expressed in flop/watt. Certainly they will do only part of the work in a complete system but still the power savings can be considerable which is very attractive these days. Bioinformatics computing can have a positive effect on our lives and the world.

When speaking of special purpose hardware, that is, computational accelerators, we should understand that they are indeed good at certain specialized computations while they may not be able to perform others. Despite the inherently parallel nature of modern computer architecture, efficiently mapping bioinformatics algorithms onto hardware resources is extremely challenging. So, not all applications can benefit from them and those which can, not all to the same degree. For example, the computations execute on external accelerators efficiently only under conditions of massive data parallelism. Programs that attempt to implement nondata parallel algorithms perform poorly.

FPGA technology has been seen by many as an ideal way to handle a lot of the data overhead in bioinformatics. We have reviewed some HMMER implementations accelerated by FPGA technology, and generally the performance is a lot better. The board can be installed in any computer, which in turn will provide a huge boost in performance. However, due to the fact that FPGAs are relatively difficult to program and optimize, there has never been any major renovations for FPGA technology within genomics.

Currently technology is moving into the spotlight as the premier technology has raised the computational challenges following the rollout of NGS instruments. This is partly due to the fact that GPUs and the popular CUDA architecture offer a rather easy way to teraflop computing. However, the bioinformatics society still expects to see implementations of known algorithms which truly exploit the cutting-edge computing technology. 

Furthermore, using accelerators effectively is not simple and trivial. Although the software tools for accelerators have improved enormously lately, for many applications it is still a big challenge to obtain a significant speedup. An important factor is that data must be transferred between the accelerator and CPU; therefore, the bandwidth of the connecting bus is a severe bottleneck in most cases. The key to high performance lies in strategies that hardware components and their corresponding software components and their corresponding software interfaces use to keep hardware processing resources busy. This can be overcome by overlapping data transport between the accelerator and host with processing. Tuning the computation and data transport task can be cumbersome. 

Whether on a compute node, cluster, grid, or cloud, today's bioinformatics software can run in parallel, executing many calculations simultaneously. Software acceleration does not require additional investment, but delivering top performance requires using threads on multiple cores or MPI on multiple nodes with SIMD. Unlike the other technologies mentioned above, SIMD is based on software acceleration, by utilizing the built-in wide instruction sets that inherently built into all x86 architecture CPUs from both Intel and AMD. There is no doubt that software acceleration, like SIMD, is cheaper, and its performance is equal to adding more hardware virtually. The fast implementation using SIMD vectorization is available in the HMMER 3 package [40]. 

Everyone in biology is now affected by parallelism. A computing cluster running accelerated application is now a competitive alternative to a traditional general-purpose cluster running serial code. Therefore, it has become a trend to emerge heterogeneous processors within a single architecture. The sign of this trend is the presence of GPGPU, FPGAs, CELL BE, and other computation accelerators in combination with standard processors. As the next generation of high-performance computing technology is coming—a heterogeneous parallel architecture, for example, the new generation of GPGPU processors which also support SIMD—a strategy of using SIMD acceleration along with cluster computing becomes even more attractive. 

In brief, we have presented a comprehensive analysis of the bottleneck of HMMER algorithm and efficiency of various implementations on modern computing platforms. Most parallel bioinformatics applications use just one of these abovementioned forms to express parallelism, but with the deeper hierarchical structure within heterogeneous computing systems, there has been a movement towards hybrid computing models. The goal of hardware acceleration or hybrid computing is to boost performance and extend the range of applications, particularly to bioinformatics computing. Hybrid computing involves combining multiple techniques together, which apply a different programming paradigm to different levels of the hierarchical system, for example, mixing MPI for internode communication with OpenMP for the intra-node parallelization. Additional parallelism can be achieved to take advantage of the distinct properties of specialized underlying hardware to gain higher-level efficiencies.

Finally, cloud computing is another way of handling the data analysis challenges, by renting access to a large CPU pool on a need-to-use basis. While Cloud Computing surely is a relatively easy and interesting alternative to setting up and running a cluster, generally cloud computing may not give real performance gains over cluster computing, because of the huge overhead of distributing the data and calculations across the leased nodes.

## 6. Conclusion

We hope that we have shown a clear trend to potential performance improvement in software and hardware acceleration combined with increased compute density, faster I/O access, and higher degree of user control over optimizations, which suggests that a heterogeneous parallel architecture makes a major breakthrough in accelerating bioinformatics applications. We expect this trend will continue in the coming years and make the bioinformatics computing roadmap more diverse and interesting.

## Figures and Tables

**Table 1:** Performance comparison among different software approaches.

Acceleration strategies	Supporting software/package	Accelerated programs	Datasets	Hardware environment	Achieved speedup
Instruction-level parallelism	SSE2 Instructions	*hmmpfam, hmmsearch *	*Pfam *and *nr* databases	2.66 GHz Intel Xeon processor with 2.5 GB of memory	1.2x~1.3x [9]

Shared memory parallelism	OpenMP	*hmmpfam *	600 HMM profiles and 250 sequences	16 x86 3.0 GHz processors, 32 MB L4 cache shared among 4 CPUs, 4 MB L3 cache, 8 GB of memory	14x [10]
	EARTH	*hmmpfam *	50 HMM profiles and 38192 sequences	A cluster that consists of 128 nodes, each with two 500 MHz Pentium III processors	222.8x [11]

Distributed memory parallelism	PVM	*hmmsearch *	1 HMM profile and 100 MB of *nr* database	A cluster with 4 nodes, each node consists of two 2.66 GHz Intel Xeon processors with 2.5 GB memory per node	4.56x [9]
	MPI	*hmmsearch *			5.90x [9]
	MPI + I/O optimizations	*hmmsearch *	One 236-state HMM profile and *nr* database	A cluster that consists of 1056 nodes, each equipped with two 3.2 GHz Intel Xeon processors, 2 GB RAM	221x [12]
		*hmmpfam *	1.6 GB of *Pfam *database and *nr* databases		328x [12]

Heterogeneous approach	MPI + SSE2	*hmmsearch *	1 HMM profile and 100 MB of *nr* database	A cluster with 4 nodes, each node consists of two 2.66 GHz Intel Xeon processors with 2.5 GB memory per node	7.71x [13]

**Table 2:** Performance comparison among different hardware approaches.

Acceleration hardware type	Hardware accelerator	Accelerated programs	Datasets	Host or base hardware environment	Reported max. speedup
Network Processor	Intel IXP 2850 network processor	*Viterbi *	Pfam_ls database (7459 models)	2.6 GHz Intel Pentium 4 CPU with 768 MB of SDRAM and 32 MB of QDR SRAM	1.82x [14]

GPGPU	8800 GTX Ultra	*hmmsearch *	3 GB nr Database (5.5 million sequences) 3 models (77, 209, 456, 789, and 1431 states)	—	38.6x [15]
	4 Tesla C1060s	*hmmsearch *	5.4 GB Database (10.54 million sequences) 3 models (128, 256 and 507 states)	2.33 GHz AMD Opteron	100+x [16]

Heterogeneous multi-core chip	CELL BE	*hmmpfam *	100 HMM states and characters	Dual-core 2.4 GHz Opteron with 8 GB RAM	~3.5x [17]

FPGA	Spartan-3 XC3S1500	*hmmsearch *	244 HMM states and a database consisting of 643,552 sequences	AMD Athlon 64 3500+	31x [18]
	Spartan-3 XC3S1500	*hmmpfam *	A database consisting of 1,544 HMMs and 1000 protein sequences	AMD Athlon 64 3500+	39x [18]
	Virtex-5 110T	*hmmsearch *	122,564 query inputs	2.33 GHz Intel Core2 Duo with 4 GB RAM	56.8x [19]

Heterogeneous approach (MPI + FPGA)	2 Spartan-3 XC3S1500	*hmmsearch *	2 HMM models (77 and 236 states) and 2 databases (217,875 and 2,521,679 sequences)	A cluster consists of 10 worker nodes, each with a dual core AMD Opteron 175 processor with 2 GB memory per node	30x [13]
